# EGFR as a biomarker of smoking status and survival in oropharyngeal squamous cell carcinoma

**DOI:** 10.1186/s40463-018-0323-6

**Published:** 2019-01-10

**Authors:** Shanmugappiriya Sivarajah, Morris Kostiuk, Cameron Lindsay, Lakshmi Puttagunta, Daniel A. O’Connell, Jeffrey Harris, Hadi Seikaly, Vincent L. Biron

**Affiliations:** 1 Division of Otolaryngology-Head & Neck Surgery, University of Alberta Hospital, 1E4, Walter Mackenzie Centre, 8440-112 Street, Edmonton, Alberta T6G 2B7 Canada; 2 Otolaryngology-Head and Neck Research Laboratory of Alberta, University of Alberta, Edmonton, Alberta Canada; 3 Department of Laboratory Medicine and Pathology, University of Alberta, Edmonton, Alberta Canada

**Keywords:** Oropharyngeal cancer, HPV, Survival, Smoking, EGFR

## Abstract

**Background:**

This study aims to investigate EGFR as a prognostic biomarker in oropharyngeal squamous cell carcinoma (OPSCC).

**Methods:**

OPSCC patients from retrospective (1998–2009) and prospective cohorts (2014–2017) were included. Retrospectively collected tumors were used to construct tissue microarrays (TMAs), which were stained with EGFR, p16, DAPI and Pan-cytokeratin, and digitally quantified. EGFR, CDKN2A and HPV E6/7 levels from prospectively collected OPSCC was measured by droplet digital PCR (ddPCR). Biomarkers were compared to patient covariates, factors and survival outcomes.

**Results:**

A total of 249 patients were included retrospectively and 64 patients were enrolled prospectively. p16 status (*p* < 0.001), smoking above 10 pack years (*p* = 0.04), smoking above 20 pack years (*p* < 0.001), total EGFR tumor levels (*p* = 0.016), and high EGFR within high or low Ki67 tumor nuclear staining (*p* = 0.03) were found to be significant predictors of 5-year disease specific survival (DSS). A Cox proportional hazard model of DSS showed smoking status and eGFR expression to be dependent of each other on predicting 5-year DSS. ddPCR analysis showed a significant association between smoking status and EGFR levels.

**Conclusions:**

Total EGFR tumor levels are predictive of 5-year DSS. EGFR levels correlate with.

smoking and could be an objective marker for this disease etiology.

**Electronic supplementary material:**

The online version of this article (10.1186/s40463-018-0323-6) contains supplementary material, which is available to authorized users.

## Introduction

High-risk human papillomavirus infection is a known cause for an increasing subset of oropharyngeal squamous cell carcinomas (OPSCC). These tumors have an epidemiologic, clinical and molecular profile that is distinct from HPV-negative OPSCC [1, 2]. Several retrospective case series have shown that HPV-positive patients have more favorable prognoses, demonstrating significantly reduced overall and disease-specific mortality rates compared to patients with HPV negative tumors [3–5].

Epidermal growth factor receptor (EGFR) is a trans-membrane tyrosine kinase receptor of the ErbB-family, that plays an important role in the development of various types of cancers. EGFR expression has been associated with several downstream pathways leading to a high tumor proliferation rate, inhibition of apoptosis, enhanced tumor invasion, and metastasis [6]. EGFR protein over-expression has been reported in 70 to 100% of head and neck squamous cell carcinomas (HNSCCs), and 46–72% of OPSCCs. In addition, EGFR gene copy number gain (EGFR gene amplification or gene high polysomy) has been detected in approximately 17–58% of HNSCCs and has been reported to be associated with a worse prognosis [7–9]. Despite this, the data on the prognostic significance of target therapies that inhibit EGFR over-expression is conflicting [7, 9–11]. Bonner et al. showed that cetuximab treatment combined with radiotherapy in loco-regionally advanced HNSCCs or with chemotherapy in recurrent/metastatic settings improved survival [12]. On the contrary, the SPECTRUM study investigating panitumumab with chemotherapy, showed a lack of significant benefit in recurrent/metastatic p16-positive OPSCCs [13]. In addition, Nakano et al. showed no correlation between EGFR protein overexpression and patient prognosis, as analyzed by chromogenic in situ hybridization in 105 cases of OPSCC [14].

The correlation between smoking and prognosis amongst HPV-positive and negative OPSCC patients has been well established in the literature [2, 3, 15]. Heavy smokers with HPV-negative disease comprise the highest risk group, with worse prognostic outcomes. Although smoking is not a strong epidemiologic co-factor in HPV-positive tumors, smoking does alter biologic behavior, rendering HPV-positive tumors less responsive to therapy [3]. In contrast, the relationship between smoking and EGFR expression in OPSCC has not been extensively investigated. Kumar et al. observed that EGFR expression was significantly higher in current smokers than in past smokers, who in turn had higher EGFR levels than those who never smoked [16]. This finding was corroborated by Baumeister et al. [6]. However, these studies did not define pack-years of tobacco smoking, include survival data, nor did they evaluate whether increased EGFR levels in smokers could be used an independent surrogate marker.

Few studies have investigated the relationship between HPV/p16 status, EGFR expression, and smoking status with survival in OPSCC patients. The purpose of this study is two-fold: 1) to establish whether EGFR expression is associated with distinct survival outcomes in p16 positive versus negative OPSCC; and 2) to determine if EGFR expression can be used as a surrogate marker for smoking positivity in OPSCC.

## Methods

### Patients

This study included two distinct cohorts of patients with OPSCC diagnosed and treated at the University of Alberta: a retrospective cohort from 1998 to 2009 from which a tissue microarray (TMA) was constructed, and a prospective cohort from 2014 to 2017 from which tissue samples were collected for ddPCR gene expression analysis. Patients from 2009 to 2014 were not included in this study because a previously construted TMA only included patients from 1998 to 2009 and prospective tissue and patient data collection did not commence until 2014. There were no notable changes in clinical practice at this institution during the excluded timespan. Patients were excluded from the study if they did not receive treatment with intent-to-cure, were lost to post-treatment follow-up, or for whom p16 status was not available (in the prospective ddPCR cohort). Further chart reviews were completed to construct databases used for further analysis including the following factors and variables: age, gender, smoking status (defined as positive with > 10 pack years and > 20 pack years) [3], pack years, dates of diagnosis and treatment, date of death, cause of death, date last known alive, treatment type, radiation type and dose, chemotherapy type and dose, tumor subsite, clinical and pathologic stage according the AJCC 7th Edition [17] and p16 status.

### Tissue microarray analysis

TMAs were constructed as previously described [2, 18, 19]. A total of 249 patient tumors were distributed over 5 TMAs, of which 218 (87.5%) had sufficient tumor tissue stained with EGFR for reliable quantification. TMAs were processed for immunofluorescence using primary antibodies specific for EGFR, p16, Ki-67, pan-cytokeratin as previously reported [19]. DAPI-containing glycerol based mounting media was used a nuclear stain. TMAs were imaged using an Aperio Scancope FL.

Digital images from TMAs were quantified using Aquanalysis to determine levels of EGFR relative to nuclear and cytoplasmic compartments in both normal and tumor containing areas of tissue cores (Additional file 1: Figure S1). Total EGFR intensity within tumor compartment areas was measured as the density of EGFR pixel intensity within the tumour compartment (includes both cytoplasm and nucleus). Ki-67 was used to calculate ratios relative to EGFR levels to take into account differences in cellularity and differentiation. Ki67 target within high intensity EGFR staining was calculated as the percent area of the total image that is occupied by tumour nuclei associated with high levels of EGFR expression. Ki67/EGFR ratios within EGFR areas was calculated as Ki67 Target in Tumor High EGFR compartment divided by Ki67 Target in Tumor Low EGFR compartment. A ratio of Ki67 in low EGFR nuclear areas was calculated as the percent area of the total image that is occupied by tumour nuclei associated with low levels of EGFR expression.

### Droplet digital PCR

RNA was extracted from tumor tissue using either the RNeasy Mini Kit (Qiagen) or the RNeasy Plus Mini Kit (Qiagen) following the manufacturer’s protocol. RNA concentration was quantified using the Qubit RNA HS assay kit (Invitrogen Cat # Q32855) on a Qubit 2.0 fluorometer as per manufacturers instructions.RNA (up to 200 ng) in a 20 ul reaction was used to synthesize cDNA using the iScript™ Reverse Transcription Supermix for RT-qPCR (BIO-RAD) and the C1000 Touch™ Thermal Cycler (catalog #185–1197 BIO-RAD) as per the manufacturer’s protocols. Following the reaction, the cDNA was diluted in nuclease free water to 0.5 ng/ul, or 1 ng/ul, and either stored at −20 °C or used directly for ddPCR.

ddPCR was carried out using the ddPCR™ Supermix for Probes (No dUTP) (BIO-RAD), the QX200™ Droplet Generator (catalog #186–4002 BIO-RAD), the QX200 Droplet Reader (catalog #186–4003 BIO-RAD) the C1000 Touch™ Thermal Cycler (catalog #185–1197 BIO-RAD) and the PX1™ PCR Plate Sealer (catalog #181–4000 BIO-RAD) as per themanufacturer’s instructions. Reactions were set up following the manufacturer’s protocols using 12 ul/reaction of 2x ddPCR Supermix for Probes (No dUTP), 1.2 ul/reaction of 20x target primers/probe for EGFR (Unique Assay ID: dHsaCPE5038080 (BIO-RAD)), 1.2 ul/reaction 20x reference primers/probe for EEF2 (Unique Assay ID: dHsaCPE5050049 (BIO-RAD)), 2.4 ul cDNA (at 0.5 ng/ul or 1 ng/ul) and 7.2 ul H2O in a 96 well plate. Reactions were mixed 3 times for 30 s at 1000 RPM using a Mixmate Vortex Shaker (Eppendorf) and 20 ul of the reaction mixture was transferred to DG8™ Cartridge for QX200/QX100 Droplet Generator (catalog #186–4008 BIO-RAD) followed by 70 μl of Droplet Generation Oil for Probes (catalog #186–3005 BIO-RAD) into the oil wells, according to the QX200 Droplet Generator Instruction Manual (#10031907 BIO-RAD). Following droplet generation, 40 ul of the reaction was transferred to wells of a 96 well plate and PCR reactions were carried out in the thermocycler using the following parameters: Step 1) 95 °C for 10 min, Step 2) 94 °C for 30 s and 60 °C for 1 min (Step 2 repeat 39 times for a total of 40), Step 3) 98 °C for 10 min and Step 4) 4 °C infinite hold. All steps had a ramp rate of 3 °C/second. Following thermocycling the reactions were read in the QX200 Droplet Reader and the RNA targets were quantified using the QuantaSoft™ Software (BIO-RAD) as previously described [20, 21]

### Cell culture

Cells were cultured and treated in 8-well plates (SCC-1, SCC-9, CAL-27, SCC-6, SCC-47, SCC-104, SCC-90 and SCC-152) and washed with 3 × 2 ml PBS, with the exception of UM-SCC-6 which were washed only 1 × 2 ml, cells were scraped, and transferred in 300 μL RNA Later (cat: AM7021, ThermoFisher Scientific) to 1.7 mL centrifuge tubes. The tubes were vortexed, and 20 μL of sample was transferred to a new centrifuge tube. RNA purification was performed using the RNeasy Plus Mini Kit supplemented with gDNA Eliminator mini Spin Columns (Cat#: 74134 Qiagen) and QIAshredder (Cat#: 79656 Qiagen) per manufacturers guidelines. RNA was used to synthesize cDNA using the iScriptTM Reverse Transcription Supermix for RT-qPCR (Cat#: 1708841 BIO-RAD) as per the manufacturer’s guidelines. Following cDNA synthesis, samples were diluted with Nuclease-free H2O to 1 ng/μl and either stored at -20 °C or used directly for droplet digital PCR (ddPCR).

### Statistical analysis

All statistical analyses were performed using SPSS version 25 (IBM, Chicaco, IL). Univariate analysis of survival was performed using the Kaplan-Meier method, with statistical significance between strata determined by the Log-Rank test. Cox-regression was used to generate univariate and multivariate hazard ratios of survival. Pearson correlation was used to examine correlations between patient variables.

## Results

### Cell culture

**Fig. 1:**
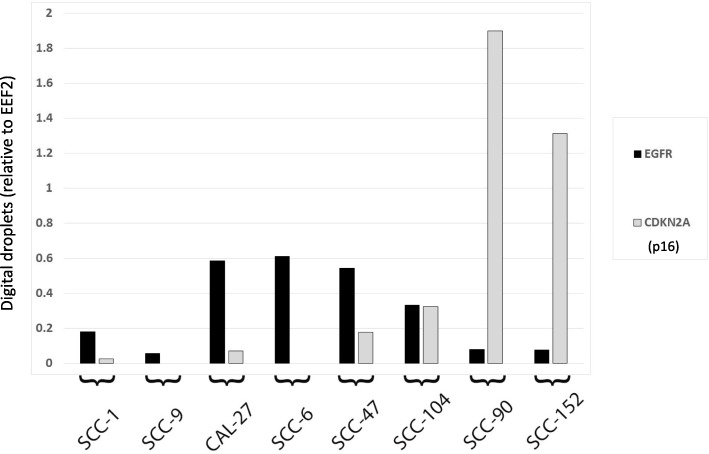
Expression of EGFR in head and neck squamous cell carcinoma cell lines. Droplet digital PCR normalized with EEF2 levels is shown for EGFR (black) and CDKN2A (grey) in several head and neck squamous carcinoma cell lines

The relationship between EGFR and p16 was initially assessed in a panel of HPV positive and negative HNSCC cell lines. Figure 1 shows EGFR and CDKN2A (gene for p16) levels in HPV positive and negative head and neck squamous cell carcinoma lines using ddPCR analysis. An inverse relationship between EGFR expression and CDKN2A levels (representative of HPV infection) is demonstrated.

### Patient characteristics

A total of 218 patients were retrospectively included for tissue microarray analysis from 1998 to 2009, and 60 patients were recruited prospectively for ddPCR analysis. The baseline characteristics of the two cohorts are listed in Table 1. Most patients were male, and there was good representation of p16 positive and negative tumors in our respective cohorts.

**Table 1:** Characteristics of patients with oropharyngeal cancer included for tissue microarray and droplet digital PCR analysis

Characteristic	TMA (*N* = 218)	ddpcr (*N* = 60)
Age (mean)	57.0	61.3
Sex (% male)	78.4	83.3
p16 positive (%)	55.5	83.3
HR-HPV POsitive (%)	–	28.3
Smoking > 10 pY (%)	72.4	56.7
Smoking > 20 py (%)	52.2	38.3
TNM stage (%)*		
I	–	15
II	–	56.7
III	23.8	18.3
IV	76.2	10
Treatment (%)		
Surgery +/− RT	58.8	81.7
RT/CRT (+/− salvage)	41.2	18.3

*TNM stage as per AJCC 7th Edition. *CRT* chemoradiation, *DDPCR* droplet digital PCR, *HR-HPV* high-risk human papillomavirus (oncogenic), *RT* radiation therapy, *TMA* tissue microarray analysis

### Survival analysis from tissue microarray

**Fig. 2:**
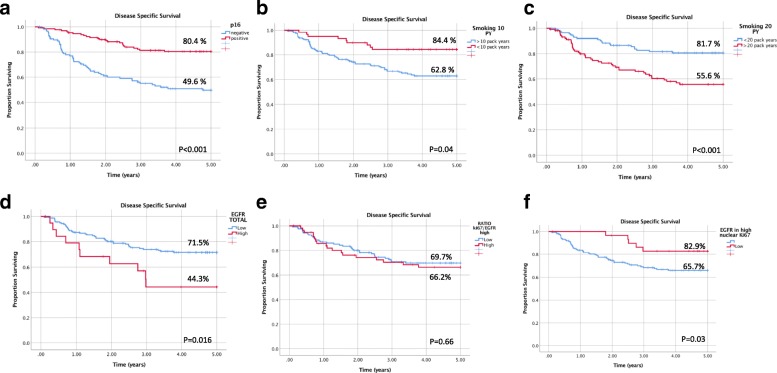
Disease specific survival in oropharyngeal cancer according to p16, smoking status and EGFR levels. Kaplan-Meier analysis of disease specific survival is shown with listed 5-year estimates when stratified by **a** p16 status, **b** smoking above 10 pack years, **c** smoking above 20 pack years, **d** total EGFR tumor levels **e** EGFR levels relative to Ki67 tumor levels and **f** high EGFR within high or low Ki67 tumor nuclear staining. *P*-value according to Log-Rank is listed at bottom right of each survival graph

In a Kaplan-Meier analysis, p16 status (*p* < 0.001), smoking above 10 pack years (*p* = 0.04), smoking above 20 pack years (*p* < 0.001), total EGFR tumor levels (*p* = 0.016), and high EGFR within high or low Ki67 tumor nuclear staining (*p* = 0.03) were found to be significant predictors of 5-year disease specific survival (DSS) (Fig. 2).

**Fig. 3:**
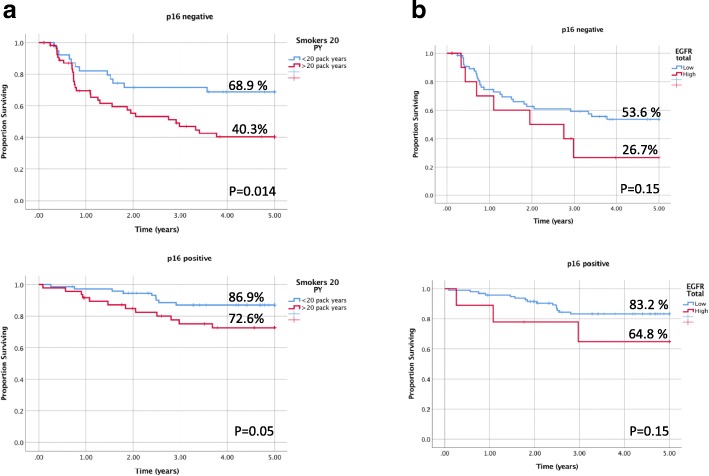
Disease specific survival of p16 positive and negative oropharyngeal cancer patients stratified by smoking and EGFR. Kaplan-Meier analysis of disease specific survival in p16 positive and negative patients is shown with listed 5-year estimates when stratified by **a**) smoking status defined by 20 pack years and **b**) total tumor EGFR levels. P-value according to Log-Rank is listed at bottom right of each survival graph

There was a significant association in 5-year DSS rates in p16 positive (*p* = 0.05) and negative (*p* = 0.014) cancers stratified by smoking status, with the lowest DSS of 40.3% observed in p16 negative patients with a greater than 20 pack-year smoking history. Although a clear trend is apparent in DSS rates, non-significant results were obtained with p16 positive (*p* = 0.15) and negative (*p* = 0.15) patients stratified by EGFR expression (Fig. 3).

A Cox proportional hazard model of disease specific survival (DSS) was performed, with univariate and multivariate analyses (Table 2). Age (HR = 1.04, 95% CI 1.01–1.07) and p16 positivity (HR = 0.36, 95% CI 0.19–0.68) were significant determinants of disease- specific survival. A univariate analysis showed significantly worse survival outcomes for 10- (HR = 2.73, 95% CI 1.35–5.54) and 20- (HR = 2.67, 95% CI 1.58–4.52) pack- year smoking histories. However, a multivariate analysis with tumor EGFR expression in combination with smoking above 10- and 20-pack years showed non-significant results for the smoking variable (*p* = 0.1 and *p* = 0.2). A similar finding was demonstrated for total EGFR levels (*p* = 0.28). A univariate analysis showed a significant association with disease specific survival, but this was not found in combination with smoking.

**Table 2:** Cox Proportional Hazard Model of Disease Specific Survival in Oropharyngeal Cancer Patients

Covariate	Univariate		Multivariate	
	Hazard Ratio (95% CI)	*P*	Hazard Ratio (95% CI)	*P*
Age	1.05 (1.03–1.08)	*< 0.001*	1.04 (1.01–1.07)	*0.01*
Gender	1.41 (0.81–2.47)	0.22	1.14 (0.59–2.20)	0.69
P16 positive	0.30 (0.18–0.51)	*< 0.001*	0.36 (0.19–0.68)	*0.001*
Smoking 10 py	2.73 (1.35–5.54)	*0.005*	1.90 (0.87–4.13) + EGFR 2.17 (1.06–4.46) - EGFR	0.1 *0.003*
Smoking 20 py	2.67 (1.58–4.52)	*< 0.001*	1.52 (0.81–2.87) + EGFR 1.69 (0.97–2.95)- EGFR	0.2 0.06
EGFR total	2.28 (1.14–4.54)	*0.02*	1.51 (0.72–3.15) + smoking 1.96 (1.0–3.91) - smoking	0.28 *0.05*
TNM stage IV (vs III)	1.07 (0.61–1.89)	0.81	1.07 (0.56–2.04)	

Multivariate models were generated using age as a continuous variable, and gender, smoking, p16 positivity, total EGFR levels and TNM stage as categorical covariates. Separate models were generated including: 1) all variables listed, 2) a model excluding smoking as shown for EGFR total and 3) models excluding EGFR for smoking status

### Correlation analysis

Pearson correlation showed significant association between smoking pack years and total EGFR (0.13, *p* = 0.046), EGFR excluding the nuclear area (013, *p* = 0.039) and p16 status (− 0.21, *p* = 0.01).

### Droplet digital PCR EGFR expression

**Fig. 4:**
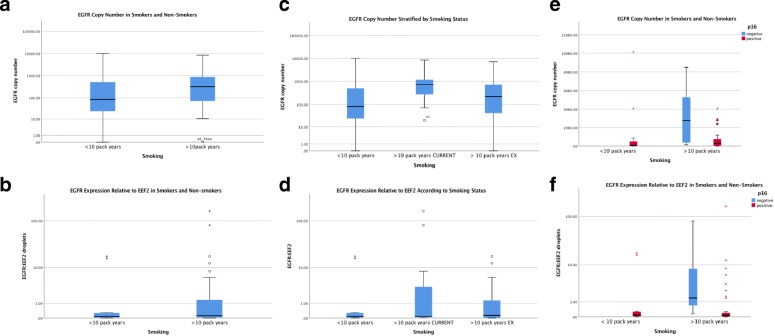
Droplet digital PCR expression of EGFR in oropharyngeal squamous cell carcinoma. EGFR levels are shown in smokers vs non-smokers according to **a** EGFR copy number and **b** EGFR gene expression. EGFR levels are further shown stratified in patients who are current smokers, ex-smokers and non-smokers by **c** copy number, **d** gene expression, **e** copy number in p16 positive vs negative patients and **f** gene expression in p16 positive vs negative patients. EGFR gene expression in shown as a value normalized with EEF2 expression

EGFR levels were detected for all prospectively included patient tissue samples. EGFR copy number was found over a broad range in patient samples but was narrowed, with mean values higher in smokers vs non-smokers (Fig. 1a). Compared to non-smokers or ex-smokers, current smokers also showed an elevated EGFR copy number (Fig. 1c). In p16 positive patients, EGFR copy number was significantly lower compared to p16 negative samples, which also displayed a broad range in values (Fig. 1e). The expression of EGFR was also measured relative to EEF2, a ubiquitous and highly expressed gene to normalize for differences in gene expression between samples. EGFR:EEF2 expression demonstrated similar results to EGFR copy number (Fig. 4b, d and f).

## Discussion

There is a growing consensus on the importance of HPV and EGFR as biomarkers or therapeutic targets in the treatment of OPSCC. Our results indicate clear associations between 1) p16 positivity with disease-specific survival; 2) total EGFR tumour levels with disease-specific survival; and 3) EGFR over-expression and smoking status. This is the first study of its kind to elucidate the prognostic significance of these biomarkers together with smoking history.

Our analysis of EGFR expression and CDKN2A levels in HPV positive and negative HNSCC cell lines showed an inverse relationship that is consistent with published studies. Although the reason why HPV-positive tumors have less EGFR expression is currently unknown, smoking has been hypothesized to be a contributory factor [20, 21]. Evaluation of ddPCR data from the current study provides more validity to this hypothesis (Fig. 4). Our results were consistent with an association between ddPCR EGFR levels and tobacco smoking (using cut-offs of 10 or 20 pack years). Previous studies have shown that tobacco smoking is a major independent prognostic factor for patients with OPSCC [2, 3, 15, 22, 23]. This finding, in relation to our results obtained through the Cox proportional hazard model of disease-specific survival lends support to the hypothesis that EGFR expression may be used as a surrogate or associative marker for smoking status in managing patients with OPSCC.

The Cox proportional hazard model revealed age, p16 positivity, 10- or 20-pack year smoking histories, and total EGFR levels as significant predictors of disease-free survival. However, our multivariate analysis revealed that a combination of smoking status with total EGFR levels is not predictive of prognostic outcomes. This indicates a significant overlap between the two, compatible with the hypothesis that EGFR expression could be used as a surrogate marker for smoking status. In the context of the limited number of studies that have previously evaluated this association, our study bears a noteworthy advantage: our data delineated specific cut-offs for pack-years of smoking to stratify patients. This is more useful than a continuum for identifying at-risk patients but will require further validation through future clinical trials.

The Kaplan- Meier analysis was supportive of the results obtained from the Cox proportional hazard model, revealing a strong inverse correlation between total tumor EGFR expression and disease-specific survival. The current data regarding the prognostic value of EGFR in OPSCC is controversial. A literature review by Bossi et al. evaluated studies that investigated both the prognostic and predictive value of EGFR in HNSCC [24]. The inconsistency in the results of studies they evaluated could be explained by heterogeneous patient cohorts with different tumor subsites, evaluation of EGFR immunoreactivity using different cut-off values, following different criteria for intensity and/or extent of the staining, as well as cytoplasmic and/or membranous staining. Our study design addressed these issues by using a homogeneous cohort of OPSCC patients, a validated EGFR antibody, TMAs with objective digital scoring of staining intensity and measurements of a different cellular compartment.

A similar inverse correlation was obtained for smoking above 10- and 20-pack years with 5-year DSS. Interestingly, our study found better discrimination in disease specific survival for p16 positive patients, when stratified for smoking above 20 pack years than 10 (*p* = 0.05 vs. *p* = 0.42) (Fig. 2, Additional file 2: Figure S2). The traditional cut-off value for smoking has been 10-pack years; our research indicates further need for evaluating these criteria to provide definitive prognostic information in p16 positive patients.

Although there was a clear trend, DSS of p16 positive and negative cancers did not statistically differ when stratified by total tumour EGFR levels. The best survival outcomes were observed in p16 positive/low EGFR patients with a DSS of 83.3%. In contrast, the worst survival outcomes were observed in p16 negative/high EGFR patients with a DSS of 26.7%. These findings are consistent with published data. Statistical significance may not have been achievable with our low sample size, with not enough power to detect a difference secondary to patient stratification.

The effects of a combination of EGFR protein expression with HPV/p16 on outcomes are poorly documented. Reimers et al. analyzed the relationship between HPV status and EGFR protein expression by immunohistochemistry (IHC) in 106 patients with OPSCC [25]. They were the first to find a trend towards an inverse relationship between EGFR expression and p16 positive OPSCC (*p* = 0.083) [20, 25]. Several studies have subsequently confirmed this inverse relationship. Hong et al. found a strong inverse association between HPV status and EGFR positivity, and showed that patients with HPV-negative/EGFR-positive cancers had an adjusted 13-fold increased risk of having loco-regional failure in comparison to patients with HPV-positive/EGFR-negative cancers [26]. However, these results have been inconsistent in the literature. Perrone et al. observed 90 patients with OPSCC, and did not find a significant difference in EGFR protein expression according to HPV status [27]. Likewise, Romanitan et al. did not find a significant difference in the expression of EGFR with HPV status [28]. In keeping with our study findings with independent prognostic markers (i.e. smoking status and EGFR expression), and ddPCR data, it is possible that HPV/p16-positive patients who smoke have a higher level of EGFR and are at particular risk of poor outcomes in comparison to their non-smoker counterparts.

We acknowledge that our study has a few limitations. This was a single-center study, with a retrospective component used for tissue microarray analysis. In addition, our study did not find a statistical significance with DSS of p16 positive and negative OPSCC patients stratified by EGFR. This may be a reflection of a diluted sample size from stratification. Our relatively smaller cohort size, especially after stratification into groups based on EGFR expression, places limitations on the widespread generalizability of this finding.

Adding total EGFR expression and HPV data to known robust clinical prognostic variables improves the prediction for survival and recurrence of disease in the pre- and post-treatment setting for patients with OPSCC. Better knowledge of tumor biology will help in classifying tumors with different prognoses, predicting response to therapy, and in enhancing therapeutic strategies to better target certain tumors. Further investigation is warranted in selecting or stratifying patients based on their biomarker profile for escalating therapy as needed.

## Conclusion

EGFR expression can be used to predict survival and is associated with smoking status in patients with oropharyngeal squamous cell carcinoma.

## Additional files

Additional file 1: Immunofluorescence staining of EGFR in cell lines, normal tissues and oropharyngeal cancer. A) In cell lines, EGFR (red) is shown in a cell line known to express EGFR (positive control) vs non-EGFR expressing cell line (negative control) relative to nuclear stain (DAPI, blue). In tissue controls, EGFR (green) is shown with or without EGFR antibody relative to nuclei (DAPI, blue). B) Normal and OPSCC cancer tissues are stained with DAPI, Pancytokeratin (PCK) and EGFR, shown as merged image in right most column (DAPI=blue, PCK=green, EGFR=red). Corresponding tissue expression values for EGFR (tAQUA) are shown. (PPTX 4928 kb)

Additional file 2: Disease specific survival according to p16 status and smoking. In p16 positive patients, smoking status does significantly influence survival when a 10 pack year cutoff is used but does in p16 negative patients. (PPTX 3660 kb)

## Abbreviations

CRT: Chemoradiation
DAPI: 4′,6′-diamidino-2-phenylindole
ddPCR: Droplet digital polymerase chain reaction
DNA: Deoxyribonucleic acid
EEF2: Eukaryotic elongation factor 2
EGFR: Epidermal growth factor receptor
HNSCC: Head and neck squamous cell carcinoma
HPV: Human papillomavirus
OPSCC: Oropharyngeal squamous cell carcinoma
PY: Pack years
RNA: Ribonucleic acid
RT: Radiation therapy
TMA: Tissue microarray
