# Supplementary figures and images for: EGFR as a biomarker of smoking status and survival in oropharyngeal squamous cell carcinoma
Source: J Otolaryngol Head Neck Surg. 2019 Jan 10;48:1. doi: 10.1186/s40463-018-0323-6 (PMC6327450; doi:10.1186/s40463-018-0323-6)

## Slide 1
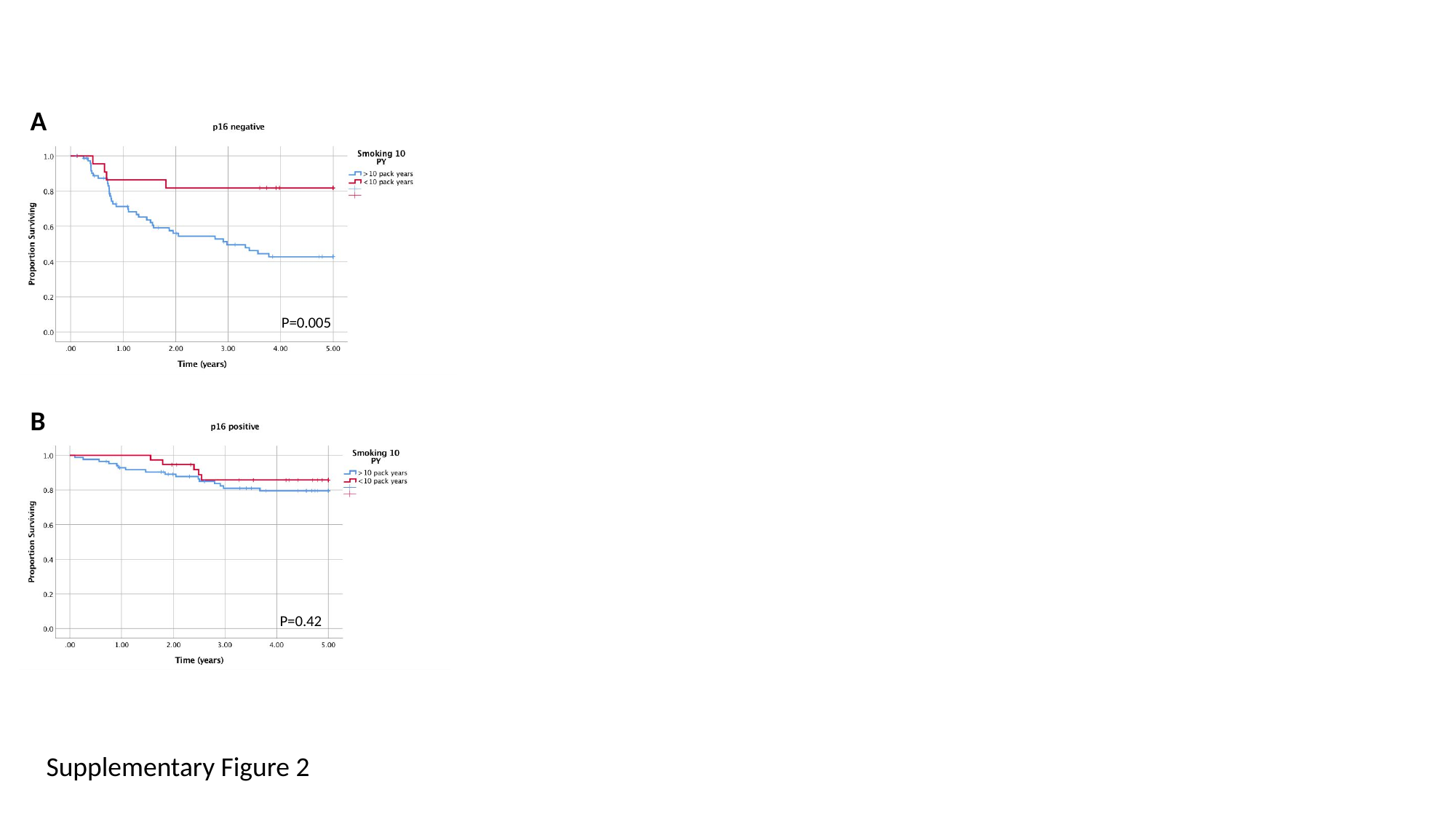

A
P=0.005
B
P=0.42
Supplementary Figure 2

Supplement: Supplementary file 2 — Disease specific survival according to p16 status and smoking. In p16 positive patients, smoking status does significantly influence survival when a 10 pack year cutoff is used but does in p16 negative patients. (PPTX 3660 kb) [file 40463_2018_323_MOESM2_ESM.pptx]
